# Forest cover percentage drives the peak biting time of *Nyssorhynchus darlingi* (Diptera: Culicidae) in the Brazilian Amazon

**DOI:** 10.1186/s12936-024-04984-1

**Published:** 2024-05-28

**Authors:** Leonardo Suveges Moreira Chaves, Eduardo Sterlino Bergo, Sara A. Bickersmith, Gabriel Z. Laporta, Jan E. Conn, Maria Anice Mureb Sallum

**Affiliations:** 1 Departamento de Epidemiologia, Faculdade de Saúde Pública, Universidade de São Paulo, Av. Dr. Arnaldo, 715 – Pacaembu, CEP, 01246-904 São Paulo, SP Brasil; 2 Instituto Pasteur, Secretaria de Estado da Saúde de São Paulo, Araraquara, SP Brazil; 3 Wadsworth Center, New York State Department of Health, Albany, NY USA; 4 Graduate Program in Health Sciences, FMABC Medical School University Center, Santo André, SP Brazil; 5 Department of Biomedical Sciences, School of Public Health, State University of New York, Albany, NY USA

**Keywords:** Mosquito behavior, Malaria, Entomological surveillance, Deforestation, Land use change

## Abstract

**Background:**

Deforestation is an important driver of malaria dynamics, with a relevant impact on mosquito ecology, including larval habitat availability, blood-feeding behaviour, and peak biting time. The latter is one of several entomological metrics to evaluate vectorial capacity and effectiveness of disease control. This study aimed to test the effect of forest cover percentage on the peak biting time of *Plasmodium*-uninfected and infected *Nyssorhynchus darlingi* females.

**Methods:**

Mosquitoes were captured utilizing human landing catch (HLC) in the peridomestic habitat in field collections carried out in the wet, wet-dry transition, and dry seasons from 2014 to 2017 in areas with active malaria transmission in Amazonian Brazil. The study locations were in rural settlements in areas with the mean annual malaria parasite incidence (Annual Parasite Incidence, API ≥ 30). All *Ny. darlingi* females were tested for *Plasmodium* spp. infection using real time PCR technique. Forest cover percentage was calculated for each collection site using QGIS v. 2.8 and was categorized in three distinct deforestation scenarios: (1) degraded, < 30% forest cover, (2) intermediate, 30–70% forest cover, and (3) preserved, > 70% forest cover.

**Results:**

The highest number of uninfected female *Ny*. *darlingi* was found in degraded landscape-sites with forest cover < 30% in any peak biting time between 18:00 and 0:00. Partially degraded landscape-sites, with (30–70%) forest cover, showed the highest number of *vivax*-infected females, with a peak biting time of 21:00–23:00. The number of *P. falciparum*-infected mosquitoes was highest in preserved sites with > 70% forest cover, a peak biting at 19:00–20:00, and in sites with 30–70% forest cover at 22:00–23:00.

**Conclusions:**

Results of this study show empirically that degraded landscapes favour uninfected *Ny*. *darlingi* with a peak biting time at dusk (18:00–19:00), whereas partially degraded landscapes affect the behaviour of *Plasmodium*-infected *Ny*. *darlingi* by shifting its peak biting time towards hours after dark (21:00–23:00). In preserved sites, *Plasmodium*-infected *Ny*. *darlingi* bite around dusk (18:00–19:00) and shortly after (19:00–20:00).

## Background

The Amazon River basin is the location of the largest tropical rainforest in the world, with the highest biodiversity and renewable water resources [1]. However, the woodland has been losing its cover to logging, extensive agribusiness, legal and illegal mining, urbanization, and construction of infrastructure [2, 3]. With continuous severe land-use change, inhabitants of these areas are constantly at risk of acquiring malaria [4]. The occurrence of malaria shows a spatiotemporal heterogeneity associated with several components that include those of the mosquito vectors, *Plasmodium* parasite, human hosts, and environment. Regarding the landscape constituents, particularly tree cover loss, forest fragmentation and other ecological factors strongly influence the mosquito vector populations [4–6].

Malaria control programmes are tasked with reducing the human infection rate using anti-malarial drugs, residual insecticides to kill the mosquito vectors, and the distribution of long-lasting insecticidal nets (LLINs) to decrease the human–mosquito contact rate [7]. However, most programmes have failed because of *Plasmodium* resistance to drugs, vector resistance to insecticides, absence of field malariology studies [8], and lack of integrated vector management [9]. The success of the latter depends on careful planning, sustainability, political support, and flexibility to reorient the programme based on robust field evidence. To build the basic control measures, it is necessary to improve knowledge of the mosquito population that will be the object of control. The peak-biting pattern is one of the crucial entomological metrics that needs to be addressed [10, 11] because ecological mechanisms leading to temporal variation within and between species are poorly understood [12] and represent the pivotal interface of human/vector contact.

*Nyssorhynchus darlingi* (formerly known as *Anopheles* (*Nyssorhynchus) darlingi*) is the primary vector of *Plasmodium vivax* and *Plasmodium falciparum* in the Amazon [13]. The peak biting activity and biting indices are some of the entomological metrics used to calculate the vectorial capacity of a mosquito vector population for monitoring the effectiveness of the control interventions. In vectorial capacity models, the biting rate (number of bites by host and time) assumes that biting is homogeneous and aggregated over time [14, 15]; therefore, close examination of the peak biting time is clearly warranted. Other entomological variables, such as mosquito abundance, density, larval habitat, climate data, seasonality, host feeding pattern, and infection rate, have been widely studied in relation to entomological control [16–18]. In addition, genetic variability appears to have an influence on multimodal blood-feeding behaviour of *Ny. darlingi* [19–23], and forest cover effect on the peak biting time of *Ny. darlingi* in areas in the Amazon River basin [24].

Malaria transmission can vary depending on both oscillations in the temperature and humidity [25] and density of mosquito vectors and *Plasmodium* parasites [16, 26, 27]. Thus, deforestation represents a major driver of malaria transmission dynamics because changes in land cover and land use are associated with microclimatic variation. In addition, environmental modifications cause changes in abiotic factors of the soil, such as pH, temperature, and solar radiation, shifting the biotic community interactions among microorganisms, plants, and animals, including mosquito diversity [28, 29]. In this new micro-ecosystem, larval habitats are the primary drivers of mosquito occurrence, increasing the abundance and fitness of dominant species [30–33]. Malaria risk can increase because of a higher probability of the rates of human–mosquito contact and boosted entomological inoculation [34, 35]. Changes in patterns of microclimatic conditions can affect mosquito longevity [36] and the extrinsic incubation period of the parasites [27]. Warmer temperatures can decrease the extrinsic incubation period of *Plasmodium*, favouring increased competence of the malaria vector population [37]. In addition, a high number of sporozoites in the female salivary glands can induce a higher biting rate, suggesting vector behaviour manipulation [38]. Other studies also show that factors such as microclimate, humidity or circadian cycle can influence mosquito behaviour in relation to blood intake and oviposition [39, 40]. Extending dry hours on various days, for example, can increase blood feeding and consequently vectorial transmission as the females attempt to survive dehydration [41]. Clearly, such a scenario could impact the biting time of parous and nulliparous females. Differences in the activity pattern of parous and nulliparous females were shown in a study conducted in the Brazilian Amazon in which a higher proportion of parous females were collected at 22:00–23:00 whereas nulliparous females were more abundant at 18:00–19:00 [22]. In contrast, a study of *Anopheles arabiensis* in southeastern Tanzania found no significant relationship between parity status and the mosquito biting time phenotype [42].

Forest cover proportion can be a relevant index for managing an effective malaria control strategy, because as a microclimate regulator it can be an indicative of mosquito biting behaviour. As an example, to attain more efficient vector control intervention and to steer the utilization and distribution of LLINs. The understanding of mosquito biting activity in general and infective and non-infective females in particular will be beneficial in comprehending the impact of using mosquito nets primarily due to the impracticality of expecting 12 h of protection. This is because during the early evening and early morning hours, people engage in activities such as cooking, eating, conversing with their families and friends, or praying outside the house [43].

## Methods

### Study sites and mosquito collection

**Fig. 1:**
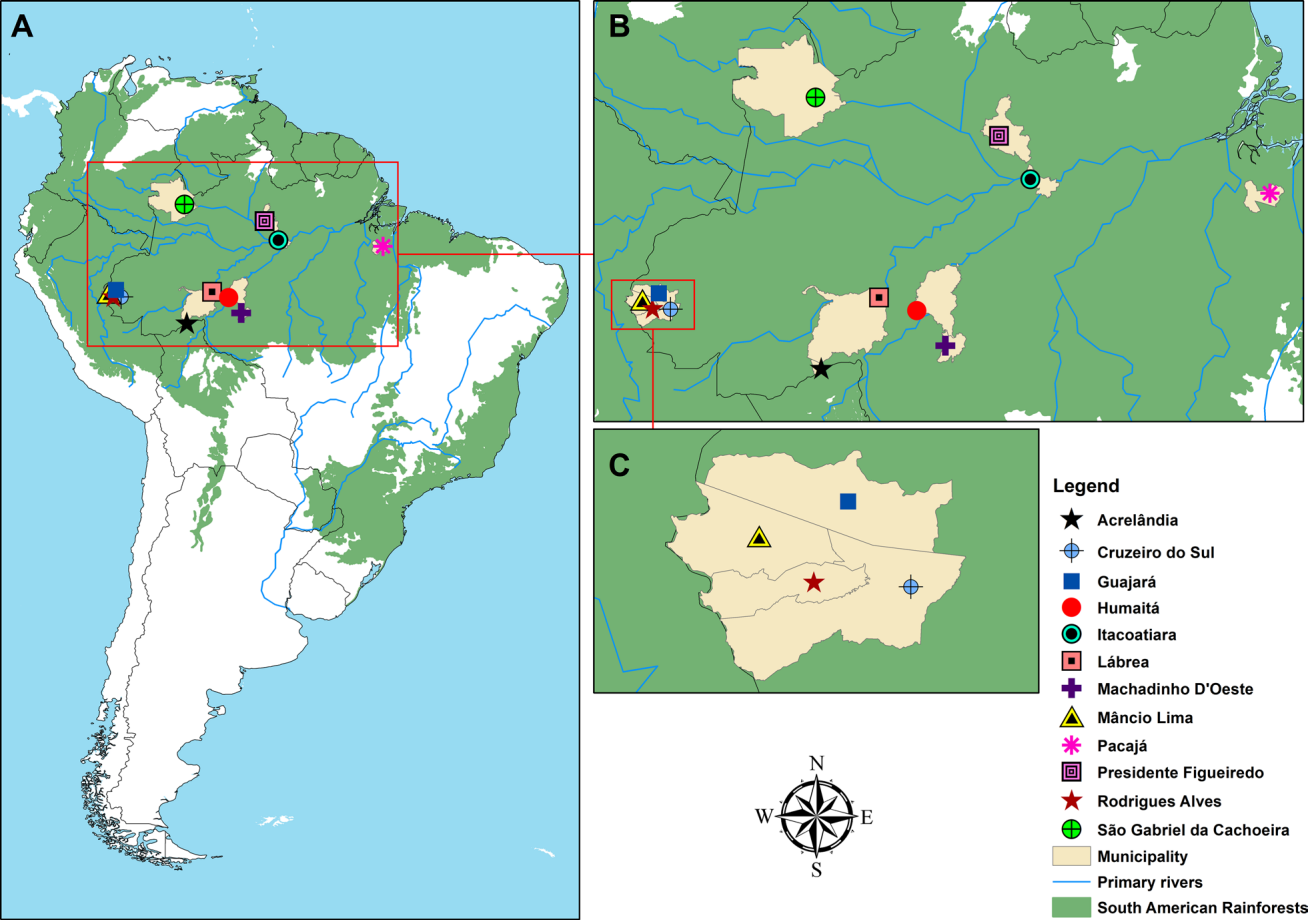
Field collection sites in 12 municipalities in the states of Acre, Amazonas, Pará and Rondônia, Brazil. **A** South America, **B** zoom-in of the studied area, **C** zoom-in of the municipalities of the Jurua River Valley

Females of the subfamily Anophelinae were collected in 80 houses in 12 municipalities in the Brazilian Amazon states of Acre, Amazonas, Pará and Rondônia states (Fig. 1; Table 1). The study locations were in rural settlements in areas with the mean annual malaria parasite incidence (Annual Parasite Incidence, API ≥ 30) of local *P. vivax* infections in the previous several months and during the period of field collections. The selection of field localities was also based on the level of forest cover, land use, and density of forest border as proxies of the presence of human and domestic animals (see [14] for additional details).

**Table 1:** Total of *Nyssorhynchus darlingi* collected in human landing catches (HLC) from 18:00 to 0:00 in the peridomestic environment of 80 landscapes sites, including *Plasmodium vivax* and *Plasmodium falciparum* infection status of females, Amazon Basin, 2015–2017

Municipality	State	Total (N)	Uninfected (n)	*P*. *vivax*-infected (n)	*P*. *falciparum*-infected (n)
Acrelândia	Acre	173	173	0	0
Cruzeiro do Sul	Acre	331	325	6	0
Guajará	Amazonas	628	617	5	6
Humaitá	Amazonas	672	671	1	0
Itacoatiara	Amazonas	74	74	0	0
Lábrea	Amazonas	1395	1379	7	9
Machadinho D’Oeste	Rondônia	896	869	24	3
Mâncio Lima	Acre	616	611	1	4
Pacajá	Pará	17	17	0	0
Presidente Figueiredo	Amazonas	3639	3639	0	0
Rodrigues Alves	Acre	761	750	8	3
São Gabriel da Cachoeira	Amazonas	1343	1310	28	5

Female adult collections were conducted from January to November, during the wet, wet-dry transition, and dry seasons. Collections were outdoors in the peridomestic environment within ~ 5 m of each of 80 houses. Houses chosen for human landing catch (HLC) were at least 2.5 km apart and were positioned in the centre of a 1 km radius circle to avoid sampling more than one house within the same 3.14 km^2^ area. The selection of field localities was based on the level of forest cover, land use, and density of forest border as proxies of the presence of human and domestic animals. All field collections were carried out solely by ESB, GZL, LSMC, and MAMS. During the field collections, the researchers were wearing protective clothes, hats, and boots. Anopheline females were collected on the legs that were covered with thick black socks to avoid mosquito bites. A hand made 12-V battery powered manual aspirator was employed to take the mosquito females from the legs before they bite. Regional climates in the study region are classified as Tropical Rainforest (Af) and Tropical Monsoon (Am) (Köppen and Geiger Classification).

HLC collections were performed one night for each of 80 houses, from 18:00 to 0:00 (Table 1). The mosquito sampling effort was 480 h-collection, distributed as follows: Acrelândia 72 h, Cruzeiro do Sul 66 h, Humaitá, Itacoatiara, Lábrea, Machadinho D’Oeste, Mâncio Lima, Pacajá, Presidente Figueiredo 36 h each, São Gabriel da Cachoeira 42 h, and Guajará, Rodrigues Alves, 24 h each. Every hour, female mosquitoes were euthanized with ethyl acetate (C_4_H_8_O_2_) vapors in the field and stored in silica gel separated by date, location, house, and collection time. Specimens were morphologically identified to species level by MAMS, labelled and stored individually with silica gel at room temperature for subsequent analysis.

## Mosquito processing

Genomic DNA was extracted from adult female *Ny. darlingi* using Qiagen DNeasy Blood & Tissue Kit (Hilden, Germany). All *Ny. darlingi* DNA samples were tested for *Plasmodium* spp. infection following [44], with DNA pools of up to five individuals containing equal amounts of gDNA. Mosquito samples with DNA concentrations of < 1.0 ng/µL or > 15 ng/µL were tested individually and not pooled. In instances where the species of *Plasmodium* could not be detected with the triplex assay, PCR amplification and agarose gel (2%) electrophoresis of PCR products was performed using primer pairs for *P. vivax* and *P. falciparum* [45]. Each PCR contained 1xPerfeCTa qPCR ToughMix, Uracil N-glycosylase (UNG), ROX (Quanta Biosciences, USA), 0.3 μM of each primer, ultrapure water, and 2 μL genomic DNA, with a total volume of 20 µL. Cycling conditions were as follows: 5 min UNG-activation hold at 45 °C and a denaturation step for 10 min at 95 °C, followed by 50 cycles of 95 °C denaturation for 15 s and 60 °C annealing/elongation for 1 min.

## Landscape sites and forest cover estimation

Each landscape site (n = 80) was centred in a house with a local family. The HLC collections were accomplished in the peridomestic environment. The forest cover percentage (0–100%) within a 1-km radius (~ 3.14-km^2^) was calculated for each landscape site using QGIS v. 2.8. Forest cover values were categorized to depict three distinct deforestation scenarios: (1) degraded, < 30% forest cover, (2) intermediate, 30–70% forest cover, and (3) preserved, > 70% forest cover, as defined by [24].

## Rationale of the study hypothesis

*Nyssorhynchus darlingi* females rest in vegetation nearby human dwellings during the day with subsequent flight to human residences at dusk (~ 18:00), when they bite humans or are captured by HLC. The study hypothesis is that the peak biting time of *Ny. darlingi* can vary according to forest cover (%) of a landscape site and status of infection of the mosquito (uninfected, *P*. *vivax*-infected, or *P*. *falciparum*-infected). The aims of the study were to test the association between forest cover percentage and (1) the peaking biting time, and (2) the number of non-infected and infected *Ny*. *darlingi* in the Brazilian Amazon.

## Models for hypothesis testing

Assessment of the number of *Ny*. *darlingi* per status of infection and biting time in a gradient of forest cover (0–100%) was undertaken using the Huisman-Olff-Fresco (HOF) multi-model selection. The models’ equations and parameters are shown in Table 2.

**Table 2:** Multi-model selection approach: models, formulas and parameters, responses, and expectations

Model	Equations and parameters	Responses	Expectations
1	$\frac{M\#}{1+{e}^{a}}$	Uniform	Null hypothesis
2	$\frac{M}{1+{e}^{a+b-x}}$	Linear	Abundance or infection correlates linearly with forest cover values
3	$\frac{M}{\left(1+{e}^{a+b*x}\right)*(1+{e}^{c})}$	Linear with Plateau	
4	$\frac{M}{\left(1+{e}^{a+b-x}\right)*(1+{e}^{c-b-x})}$	Unimodal	Abundance or infection has a unimodal correlation with forest cover values
5	$\frac{M}{\left(1+{e}^{a+b*x}\right)*(1+{e}^{c-d*x})}$	Asymmetric Unimodal	
6	$\frac{M}{\left(1+{e}^{a+b*x}\right)*(1+{e}^{c-b*x})}+\frac{M}{\left(1+{e}^{a+b*(x-d)}\right)*(1+{e}^{c-d*(x-d)})}$	Bimodal	Abundance or infection has a bimodal correlation with forest cover values
7	$\frac{M}{\left(1+{e}^{a+b*x}\right)*(1+{e}^{c-b*x})}+\frac{M}{\left(1+{e}^{a+b*(x-d)}\right)*(1+{e}^{c-f*(x-d)})}$	Asymmetric Bimodal	

Models were assumed having Poisson errors–Poisson regressions were worked out

^#^*M* is the maximum abundance or infection value which is within the positive integer set (1, 2, 3,…, *n*) for Poisson data. Model variables a–d and f are optimized in the process of model fitting [46]

Comparisons of total numbers of *Ny*. *darlingi* that were uninfected, *P*. *vivax*-infected, and *P*. *falciparum*-infected along this gradient were carried out at six different hour-long periods: 18:00–19:00; 19:00–20:00; 20:00–21:00; 21:00–22:00; 22:00–23:00; and 23:00–0:00. Regression curves were fitted to these data employing maximum likelihood estimates, the Akaike Information Criteria corrected for small samples (AICc), and bootstrap model checking. All analyses were run in *R* v. 4.3 (R Development, Core Team, Vienna, Austria). This approach has been successfully employed to test the intermediate disturbance hypothesis with mosquito community data in a gradient of forest cover in Panama [47]. Means and standard deviations per forest cover categories (< 30%, degraded; 30–70%, intermediate; and > 70%, preserved) were presented along with the regression curves.

## Results

### Study sites and anopheline collection

**Fig. 2:**
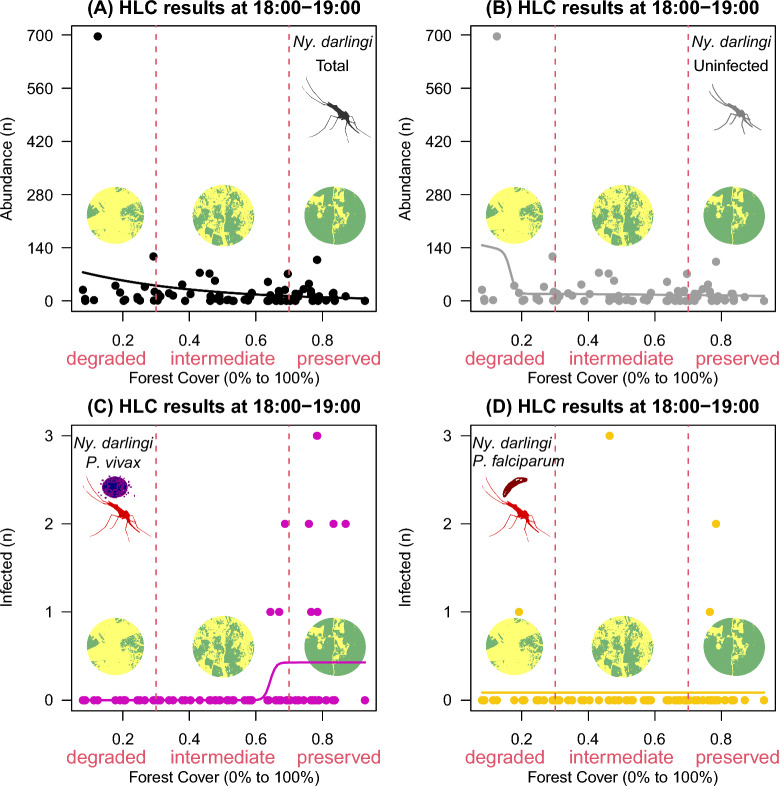
HOF multi-model selection scheme showing the fitted curve to *Nyssorhynchus darlingi* females sampled by human landing catch (HLC) at 18:00–19:00, according to infection status, along a gradient of forest cover (0–100%) across the 80 landscape sites

**Fig. 3:**
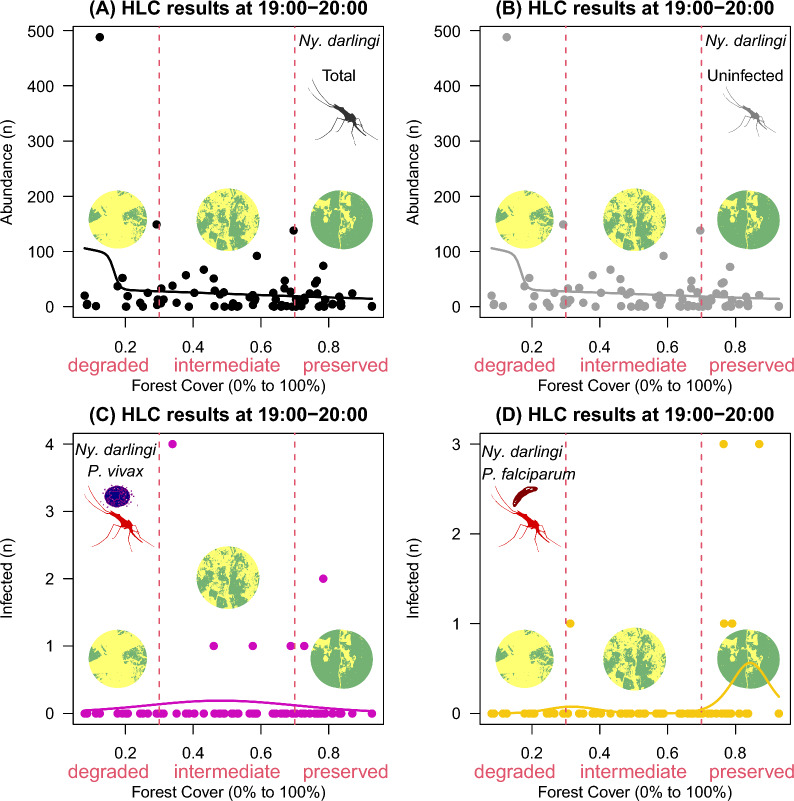
HOF multi-model selection scheme showing the fitted curve to *Nyssorhynchus darlingi* females sampled by human landing catch (HLC) at 19:00–20:00 according to infection status along a gradient of forest cover (0–100%) at landscape sites

**Fig. 4:**
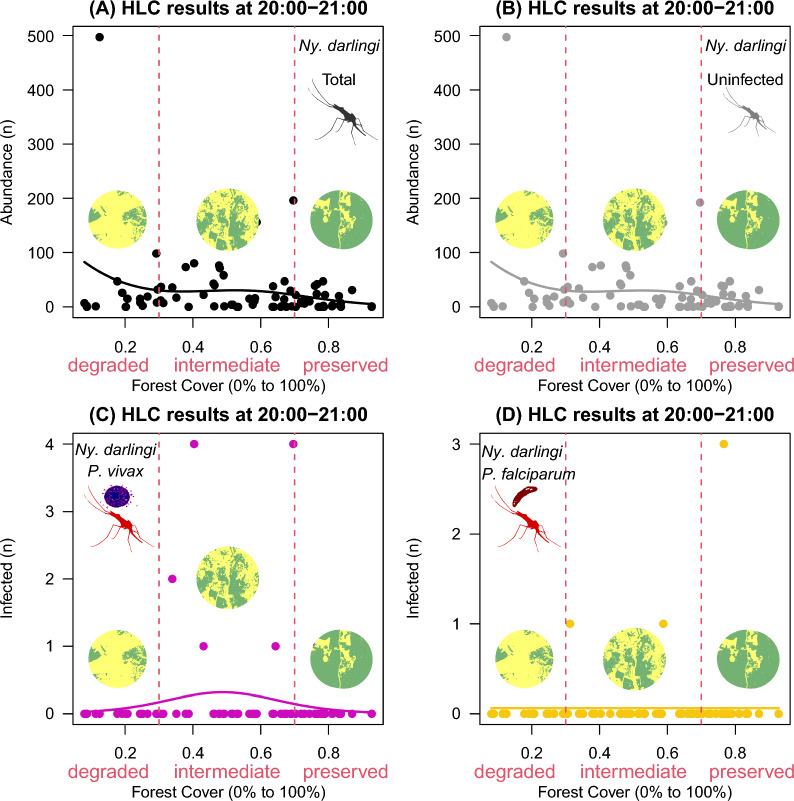
HOF multi-model selection scheme showing the fitted curve to *Nyssorhynchus darlingi* females sampled by human landing catch (HLC) at 20:00–21:00 according to infection status along a gradient of forest cover (0–100%) in landscape sites

**Fig. 5:**
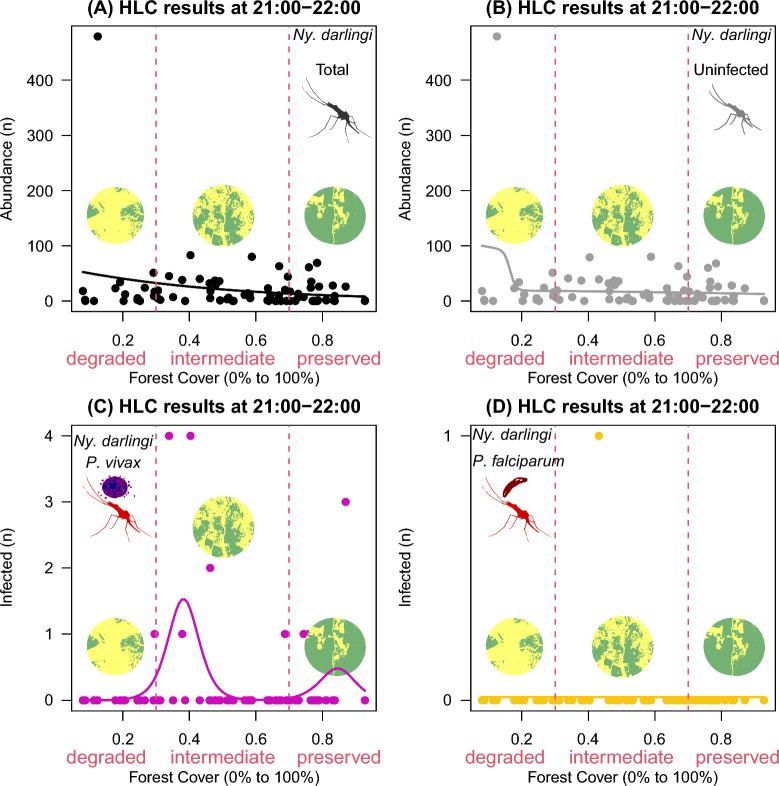
HOF multi-model selection scheme showing the fitted curve to *Nyssorhynchus darlingi* females sampled by human landing catch (HLC) at 21:00–22:00 according to infection status along a gradient of forest cover (0–100%) in landscape sites

**Figure. 6:**
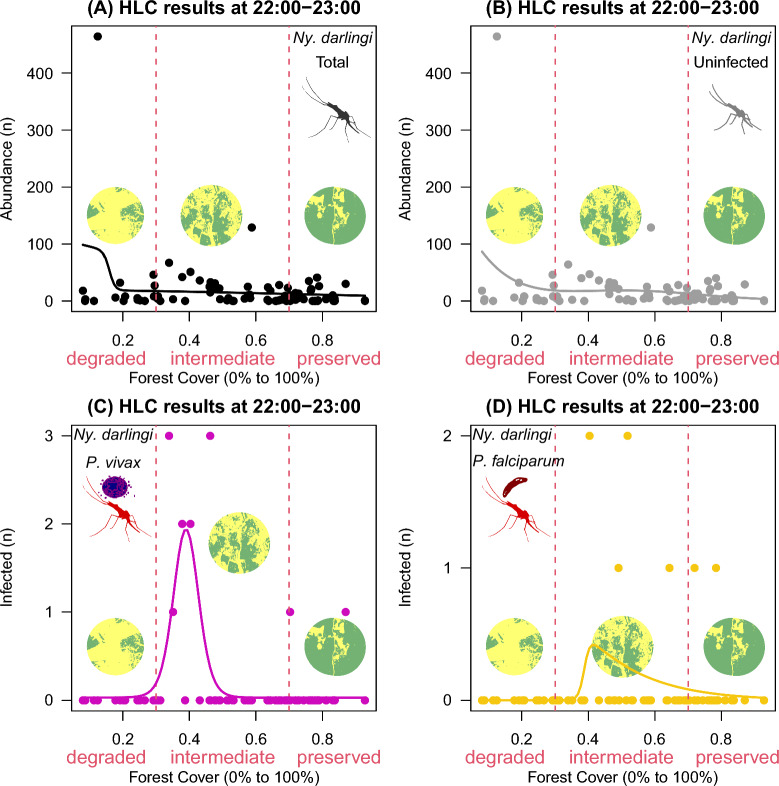
HOF multi-model selection scheme showing the fitted curve to *Nyssorhynchus darlingi* females sampled by human landing catch at 22:00–23:00 according to infection status along a gradient of forest cover (0–100%) in landscape sites

**Fig. 7:**
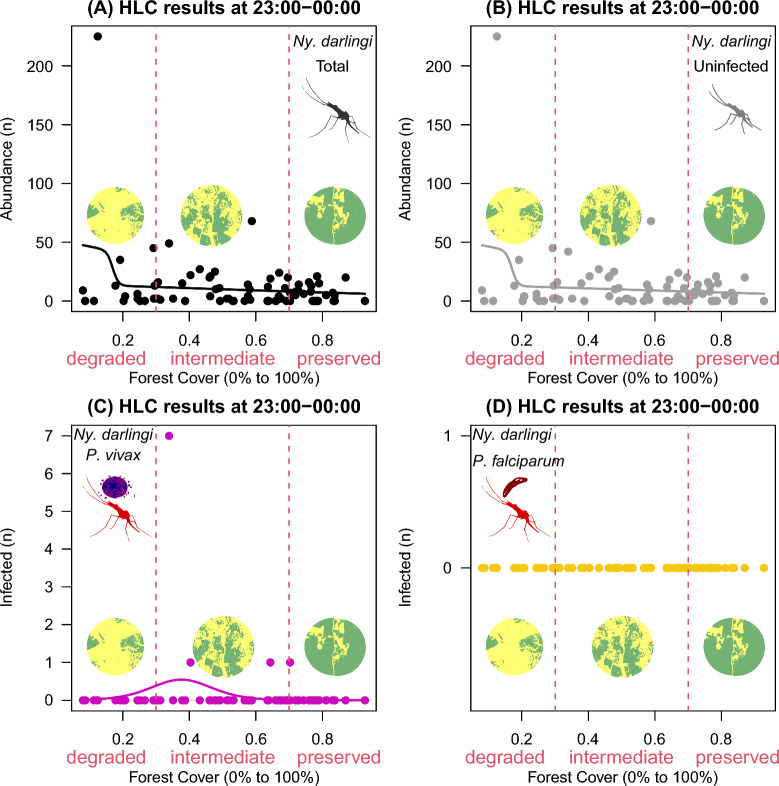
HOF multi-model selection scheme showing the fitted curve to *Nyssorhynchus darlingi* females sampled by human landing catch at 23:00–00:00 according to infection status along a gradient of forest cover (0–100%) in landscape sites

A total of 10,545 *Ny*. *darlingi* females were collected between 18:00 and 00:00 in 80 landscape sites in which forest cover varied from 8 to 93%. This total was composed of three groups of *Ny*. *darlingi* females: uninfected (10,435; 99%); infected with *P*. *vivax* (80; 0.75%); or with *P*. *falciparum* (30; 0.25%). In Figs. 2, 3, 4, 5, 6 and 7, the closed circles represent landscape-sites (n = 80) where outdoor HLC were performed, i.e., in panel A is shown the distribution of total abundance of *Ny*. *darlingi* collected per landscape-site, in panel B, the distribution of the uninfected fraction, in panel C, the *P*. *vivax*-infected and in panel D, the *P. falciparum*-infected. The black line represents the fitted curve from the best statistical model to the total *Ny*. *darlingi* abundance, the gray line is the curve from the best statistical model to the uninfected fraction, and analogously, magenta, and yellow curves represented the fitted statistical models to the infected *P*. *vivax*- and *P. falciparum*-*Ny*. *darlingi* fractions.

Overall, the uninfected female fraction had a different peak biting time compared with the infected counterparts. At 18:00–19:00, HLC yielded more infected and uninfected females (Fig. 2A) and more uninfected *Ny*. *darlingi* females in landscape sites with < 30% forest cover (62 ± 172 in degraded vs. 16 ± 22 at intermediate vs. 14 ± 21 in preserved; Fig. 2B). This difference was caused by a single observation, a collection made in one landscape-site in Presidente Figueiredo County, Amazonas state (Fig. 2B). If this observation is removed, the uninfected *Ny*. *darlingi* abundance was similar at any forest cover percentage. However, *P*. *vivax*-infected females were found in locations with > 70% forest cover (0 ± 0 in degraded vs. 0.1 ± 0.4 at intermediate vs. 0.4 ± 0.9 in preserved) (Fig. 2C), whereas *P. falciparum*-infected females were collected in small number in any forest cover percentage (Fig. 2D).

At 19:00–20:00, total females (Fig. 3A) peaked at degraded locations, *P*. *vivax*-infected *Ny*. *darlingi* females peaked at intermediate (Fig. 3C; 0 ± 0 in degraded vs. 0.2 ± 0.7 at intermediate vs. 0.1 ± 0.4 in preserved), and *P*. *falciparum*-infected at conserved (Fig. 3D; 0 ± 0 in degraded vs. 0.03 ± 0.16 at intermediate vs. 0.3 ± 0.8 in preserved) portions of the forest cover gradient, whereas the uninfected fraction showed a higher frequency at the degraded portion of this gradient ( 52 ± 122 in degraded vs. 26 ± 36 at intermediate vs. 13 ± 17 in preserved; Fig. 3B).

*Nyssorhynchus darlingi* females infected with *P*. *vivax* exhibited higher frequency levels at the intermediate portion (0 ± 0 in degraded vs. 0.3 ± 1 at intermediate vs. 0.04 ± 0.2 in preserved; Fig. 4C) of forest cover gradient at 20:00–21:00. No obvious pattern was seen for those infected with *P*. *falciparum* during this period (Fig. 4D). Total females (Fig. 4A) and uninfected females were found throughout the gradient, particularly in the degraded portion (49 ± 122 in degraded vs. 30 ± 42 at intermediate vs. 12 ± 14 in preserved; Fig. 4B).

A bimodal curve for intermediate and conserved portions of the forest cover gradient was seen for *Ny*. *darlingi* infected with *P*. *vivax* at 21:00–22:00 (0.06 ± 0.3 in degraded vs. 0.3 ± 1 at intermediate vs. 0.2 ± 0.7 in preserved; Fig. 5C). Total females (Fig. 5A) and uninfected females occurred in all forest cover levels (Fig. 5B), and *P*. *falciparum* infected females (Fig. 5D) showed no clear pattern.

Females infected with both *P*. *vivax* and* P*. *falciparum* showed specific responses at 22:00–23:00 peaking at the intermediate forest cover (0.0 ± 0.0, 0.0 ± 0.0 in degraded vs. 0.3 ± 0.8, 0.2 ± 0.5, at intermediate vs. 0.08 ± 0.3, 0.08 ± 0.3 in preserved; Fig. 6C, D). Total females (Fig. 6A) and uninfected females (Fig. 6B) had the same pattern as seen for the previous biting times (Figs. 2–5).

Lastly, *P*. *vivax*-infected females peaked at the intermediate forest cover in 23:00–00:00 (0 ± 0 in degraded vs. 0.2 ± 1 at intermediate vs. 0.04 ± 0.2 in preserved; Fig. 7C). No females infected with *P*. *falciparum* were found (Fig. 7D) and total (Fig. 7A) and uninfected females again showed no specific response to forest cover levels although there was an association with degraded landscape sites (Fig. 7B).

## Discussion

Deforestation, forest fragmentation and the percentage of the forest cover in areas with endemic malaria transmission are key drivers of the density of species of Anophelinae that are *Plasmodium* vectors, increasing the likelihood of human exposure to infected mosquito bites, intensity of *Plasmodium* transmission, and spread of malaria [4, 48, 49]. Results of the current investigation, focused on the effects of tree cover loss on the biting behaviour of *Ny*. *darlingi,* showed that forest cover percentage can affect the peak biting time of this species across the Amazon River basin. The blood feeding behaviour of *Ny*. *darlingi* has been studied [12, 14, 50], and it is known that it has multimodal peaks [19, 21, 22], and usually exophilic biting behaviour [51], associated with environmental variables [52]. Recently, Oliveira et al*.* [24] determined that in rural settlements with high edge density and forest cover between 30 and 70%, the prevalence of Anophelinae mosquitoes is high, and the number of *Ny*. *darlingi* decreased across the 12-h collections from high abundance in the early evening to the lowest, in the early morning, between 03:00 and 06:00. In addition, the number of *Plasmodium* infected mosquitoes was significantly higher from midnight to 03:00 than from 18:00 to 21:00, 21:00 to 00:00, 03:00 to 06:00.

In the present study, the uninfected population of *Ny. darlingi* also demonstrated a different peak biting time compared to the infected populations. From 18:00 to 19:00, the HLC collections yielded a higher proportion of uninfected *Ny*. *darlingi* females in degraded areas with ~ 20% forest cover, whereas *P*. *vivax*-infected females were found in partially degraded areas with > 60% forest cover. *Plasmodium vivax* and *P*. *falciparum*-infected *Ny*. *darlingi* females peaked in areas with intermediate and high forest cover percentage, whereas the uninfected females showed higher frequency at the degraded portion of this gradient from 19:00 to 20:00.

Despite the evidence of the study’s findings, further investigations are needed to verify and quantify the influence of the percentage of forest cover and fragmentation on mosquito peak biting behaviour, and on the dynamics of *Plasmodium* transmission. Other variables that can influence mosquito community composition and life history, such as thermic amplitude of the soil surface, soil microbiome, and temperature of freshwater ecosystems in varied forest cover need to be investigated to understand any substantial impact they may exert on peak biting time of malaria vectors. Forest cover can influence the local microclimate by increasing the range of daytime temperature and humidity, variables essential to mosquito biting profiles. Recently, an investigation focusing on *Anopheles farauti* showed that temperature is an important predictor of the biting activity in Australia. Besides temperature, light intensity, humidity, collector, and season are important predictors. Despite being non-linear, the temperature had a positive effect on the female biting activity, whereas the impact of humidity was more complex [53]. Because deforestation decreases tree evapotranspiration, leading to changes in both the microclimate factors and seasonality of dry and wet seasons in the Amazon tropical rain forest [54], variation detected in the peak biting time of infected and non-infected females in a gradient of forest cover percentage may be linked to variation in temperature and humidity during the night. It is also important to consider that this study of *Ny. darlingi* biting activity was conducted across the wet, wet-dry transition, and dry seasons, thus the peak biting profile of infected and non-infected females, might also differ seasonally; furthermore, other infected/non-infected mosquito species might respond differently.

The study’s observation in rural settlements in the Amazon provide support for an association between the number of non-infected *Ny*. *darlingi* and low forest cover, whereas landscapes with > 75% of forest cover are associated with high numbers of infected mosquitoes. The latter finding can be explained by the high incidence of human malaria in the local inhabitants [24], likely because they have poor access to health facilities, delay in malaria diagnosis and anti-malarial treatment such as that observed in areas of frontier malaria [55]. In landscapes with > 75% forest cover, infected females peaked from 19:00 to 20:00 in the peridomestic environment when humans are generally still awake. Several factors may help to understand this peak biting time of infected females, for example, parous females may rest on the forest edge vegetation to digest blood and develop eggs, and the human dwellings are adjacent the forest edge, an easy source of a subsequent blood meal. It is also necessary to investigate whether differences in peak biting times have a genetic basis as demonstrated in Mâncio Lima, Acre state, Brazilian Amazon for *Ny*. *darlingi* [23]. In the northeastern Brazilian Amazon, a high frequency of parous *Ny. darlingi* females was collected from 20:00 to 22:00 whereas nulliparous females were more abundant from 18:00 to 19:00. Unfortunately, information on forest cover percentage and forest fragmentation is unavailable from this study in the district of Coração in the outskirts of Macapá, Amapá state, to compare potential associations among parity rate, peak biting time and forest cover [22].

Changes in biodiversity have been reported to increase malaria incidence [56–58]. Anthropogenic modifications in natural environments can cause changes in the richness and distribution of species [32], shifts in the circadian clock of a mosquito vector [59], and impact behaviours of a species: anthropophilic/zoophilic and exophagic/endophagic [51]. In areas with high mosquito diversity, diffuse competition for hosts can negatively impact blood feeding activity [60]. Therefore, the abundance of non-vector species can modify the peak temporal activity of *Ny*. *darlingi*, and diffuse competition can support this process.

Synergism among low forest cover, biodiversity loss, and changes in the local climate can shift the peak biting time of mosquitoes, as in species of the tribe Aedini and other groups in the Atlantic Forest [61]. Also, seasonality modulates the behaviour of the African malaria vector, *An*. *arabiensis*, shifting the biting preference from indoors to outdoors [62]. In the Amazon River basin, the density of malaria vectors depends on the annual seasonal cycle of rainfall. Both high precipitation and cooler temperatures during the rainy season and higher temperatures and lower precipitation in the dry season affect mosquito abundance [18, 63]. In this context, both environmental and landscape factors can influence on *Ny*. *darlingi* behaviour [17, 24]. In Blondin village in the Oyapock River, Amazonian French Guyana, Vezenegho et al*.* [64] found that a statistically significant higher percentage of *Ny. darlingi* was collected between 20:30 and 22:30 compared to those caught from 18:30 to 20:30 and from 05:00 to 07:00 at the long dry season. Despite the difference observed in *Ny. darlingi* peak biting time in the long dry season, there was no difference in the biting peaking time during the short rainy season.

In areas where *Ny*. *darlingi* is the dominant vector, the risk of malaria can be influenced by the control measures adopted, the vector species biting profile, and the plasticity in the blood feeding behaviour of the vector females [50, 65]. In addition, knowledge of the proportion of nulliparous or multiparous females can indicate the density of females that can carry *Plasmodium* parasites because of previous feeding on the blood of infective human [22]. This information can be a significant contribution to evaluate vector control strategies focused on decreasing malaria transmission.

The results of the current investigation revealed that areas with about 40% forest cover from 22:00 to 23:00, and localities with 83% forest cover from 19:00 to 20:00, had a higher proportion of *P. vivax* and *P. falciparum* infected *Ny. darlingi*. Conversely, a higher number of uninfected females was found in areas with less than 25% forest cover during all time slots examined. The link between percentage of forest cover and peak biting activity of infected and non-infected *Ny. darlingi* in the Amazon is supported by the results of the study. Additionally, the peak biting time of both *P. vivax* and *P. falciparum* infected and non-infected females varies depending on the time slot and forest cover percentage. These discoveries present an added hurdle for malaria control programmes because there is strong evidence that *Ny. darlingi* can transmit *P. vivax* and *P. falciparum* outdoors during the night, at different hours between 18:00 to midnight, depending on the percentage of forest cover.

## Conclusions

In this study, the biting peak time of female *Ny. darlingi* infected and uninfected with *Plasmodium* was examined across a range of forest cover percentages in the peridomestic environment in rural settlements across Brazilian Amazon. *Nyssorhynchus darlingi* females can bite from 18:00 to midnight in all ranges of forest cover investigated. The proportion of *P. vivax* and *P. falciparum* infected and uninfected females fluctuated with the time of collection and forest cover percentage. The level of deforestation in the landscape affects the biting peak time of the infected females of *Nyssorhynchus darlingi*. Consequently, the temporal dynamic of infected females in the landscape is a key factor to looking for more effective control/elimination methods.

## Data Availability

The datasets used and/or analysed during the current study are available from the corresponding author on reasonable request.

## Abbreviation

HLC: Human landing catch collection
